# Secondary sclerosing cholangitis: an unusual presentation of leptospirosis

**DOI:** 10.1186/s41182-025-00700-0

**Published:** 2025-02-12

**Authors:** Tilan Aponso, W. M. D. A. S. Wanninayake, I. P. Wijesinghe, Nethma Jayasekara, Waruna Iddamalgoda, W. M. M. A. Wanasinghe

**Affiliations:** 1 Gastrointestinal and Hepatology Unit, Army Hospital, Colombo, Sri Lanka; 2 Medical Unit, National Hospital of Sri Lanka, Colombo, Sri Lanka; 3 Pathology Unit, Army Hospital, Colombo, Sri Lanka

## Abstract

Sclerosing cholangitis is a rare progressive cholestatic disease that is classified as secondary sclerosing cholangitis when it is caused by an identifiable cause. Sclerosing cholangitis has been linked to infections like COVID-19 and parasitic infections like Clonorchis sinensis and Ascaris lumbricoides. However, leptospirosis has not been linked to sclerosing cholangitis in the medical literature. In this article, we report a 37-year-old gentleman who was diagnosed with leptospirosis, worsened by painless cholestasis, while he was improving from leptospirosis. Magnetic resonance cholangiopancreatography revealed multiple short-segment biliary strictures, segmental dilatation, and mural irregularities in both intrahepatic ducts confirming the diagnosis of sclerosing cholangitis. After ruling out other potential causes and considering the initial presentation during a leptospirosis infection, we concluded that leptospirosis caused secondary sclerosing cholangitis. We report this as the first case of secondary sclerosing cholangitis in a leptospirosis patient without renal, respiratory, or cardiac complications, emphasizing the importance of ruling out this cause in a leptospirosis patient with persistent cholestasis.

## Introduction

Sclerosing cholangitis is a rare progressive cholestatic disease that can affect both intra- and extra-hepatic biliary ducts. It is characterized by stricturing, beading, and fibrosis of the biliary ducts. Sclerosing cholangitis is classified into primary and secondary sclerosing cholangitis. Secondary sclerosing cholangitis arises from identifiable causes, such as biliary tract infections, ischemic biliary injury, drugs, toxins, or inflammatory processes. However, we classify it as primary sclerosing cholangitis when the cause is idiopathic [1, 2]. Primary sclerosing cholangitis has an incidence range from 0 to 1.3 per 100,000 persons/year, and prevalence rates are 0 to 16.2 per 100,000 persons [3]. Secondary sclerosing cholangitis is a relatively uncommon disease compared to primary sclerosing cholangitis. Studies have not provided a clear understanding of the prevalence and demographics of secondary sclerosing cholangitis.

Secondary sclerosing cholangitis has been associated with several types of infections. These include bacterial cholangitis from Escherichia coli, Klebsiella pneumoniae, and Enterococcus spp. [4]; clonorchiasis [5]; amoebic cholangitis [6] from Entamoeba histolytica; Mycobacterium tuberculosis [7]; cytomegalovirus and cryptosporidiosis [8]; and parasitic infections like Schistosoma mansoni and Schistosoma japonicum [9]. COVID-associated cholangiopathy after severe COVID-19 infection has also been implicated in secondary sclerosing cholangitis in the COVID-19 pandemic [10]. However, the medical literature does not report leptospirosis without renal, respiratory, or cardiac complications as the cause of sclerosing cholangitis.

## Case presentation

A 37-year-old gentleman presented with fever, headache, myalgia, and conjunctival suffusion for 4 days. He provided a detailed history of significant mud exposure two weeks prior to his presentation. He was perfectly healthy before this admission.

On examination, he was febrile and tachycardic with a pulse rate of 110 bpm and blood pressure of 110/70 mmHg. He was icteric and had tenderness in his right hypochondrial area. However, the examination did not reveal a hepatosplenomegaly. He also had normal respiratory and neurological examination findings.

Due to a clinical suspicion of leptospirosis, we started him on intravenous ceftriaxone 2 g daily after withdrawing blood samples for basic and specific investigations. Table 1 demonstrates the investigations that the patient had undergone during his clinical course. The patient had a favorable clinical response to ceftriaxone, and he was fever-free on day 5. On the other hand, even though he was not on hepatotoxic drugs, his painless jaundice began to worsen after Day 7. He underwent extensive investigations for cholestasis (Table 1). He was started on Ursodeoxycholic acid 15 mg/kg dose and added Cholestyramine 4 g twice daily. Naltrexone and sertraline were also added later due to devastating cholestatic symptoms.

**Table 1:** Investigations

Investigations with normal values	Day 3	Day 7	Day 14	Day 60
White cell count	18 × 10^3^	11 × 10^3^	9 × 10^3^	9 × 10^3^
Hemoglobin	12 g/dL	12.5 g/dL	12.5 mg/dL	12.5 mg/dL
Platelet count	163 × 10^3^	246 × 10^3^	255 × 10^3^	253 × 10^3^
C-reactive protein (< 6 mg/dL)	163 mg/dL	35 mg/dL	10 mg/dL	< 6 mg/dL
Creatinine level (0.7- 1.3 mg/dL)	1 mg/dL	1 mg/dL	1 mg/dL	1 mg/dL
Aspartate aminotransferase (< 50U/L)	85U/L	108U/L	38U/L	37U/L
Alanine transaminase (< 50U/L)	89U/L	170U/L	42U/L	44U/L
Direct bilirubin (0–24 umol/L)	87.21 umol/L	376.2 umol/L	689.1 umol/L	171 umol/L
Alkaline phosphatase (< 147 IU/L)	238 IU/L	578 IU/L	1100 IU/L	266 IU/L
Gamma-glutamyl transferase (40 U/L)	64U/L	135U/L	534U/L	96U/L
Serum albumin (3.5—5.2 g/dl)	3.8 g/dl	3.8 g/dl	4.0 g/dl	4.8 g/dl
International normalized ratio (< 1.2)	1.1	1.4	1.3	1
Abdominal ultrasound	Mild hepatomegaly without biliary duct dilatation Kidney, gall bladder, and pancreas appear normal			
Blood, urine, and sputum culture	Negative			
Chest x-ray	Normal			
Leptospira IgM	Positive			
Leptospira IgG	Negative			
Microscopic Agglutination Test (MAT)	Positive (≥ 1:320)			
Hepatitis B surface antigen	Negative			
Hepatitis C antibody	Negative			
HIV screening	Negative			
Antinuclear Antibody	Negative			
Anti-mitochondrial antibody	Negative			
Cytomegalovirus IgM	Negative			
Antineutrophil cytoplasmic antibodies	Negative			
Angiotensin converting enzyme level	Negative			
Tuberculosis screening	Negative			
Stool calprotectin	Negative			
Gastroscopy and colonoscopy	Negative for inflammatory bowel disease			
Magnetic resonance cholangiopancreatography on day 12	Multiple short-segment biliary strictures, segmental dilatation, and mural irregularities in both intrahepatic ducts suggestive of early primary sclerosing cholangitis.(Fig. 1) Kidney, gallbladder, and pancreas appear normal			
Liver histology on day 14 (Fig. 3)	Patchy ductular inflammation with lymphocytes, plasma cells, and scattered neutrophils. Panlobular, intracytoplasmic, and canalicular cholestasis present Mild mixed inflammation is evident in portal tracts No interphase hepatitis, iron, or copper deposits

**Fig. 1:**
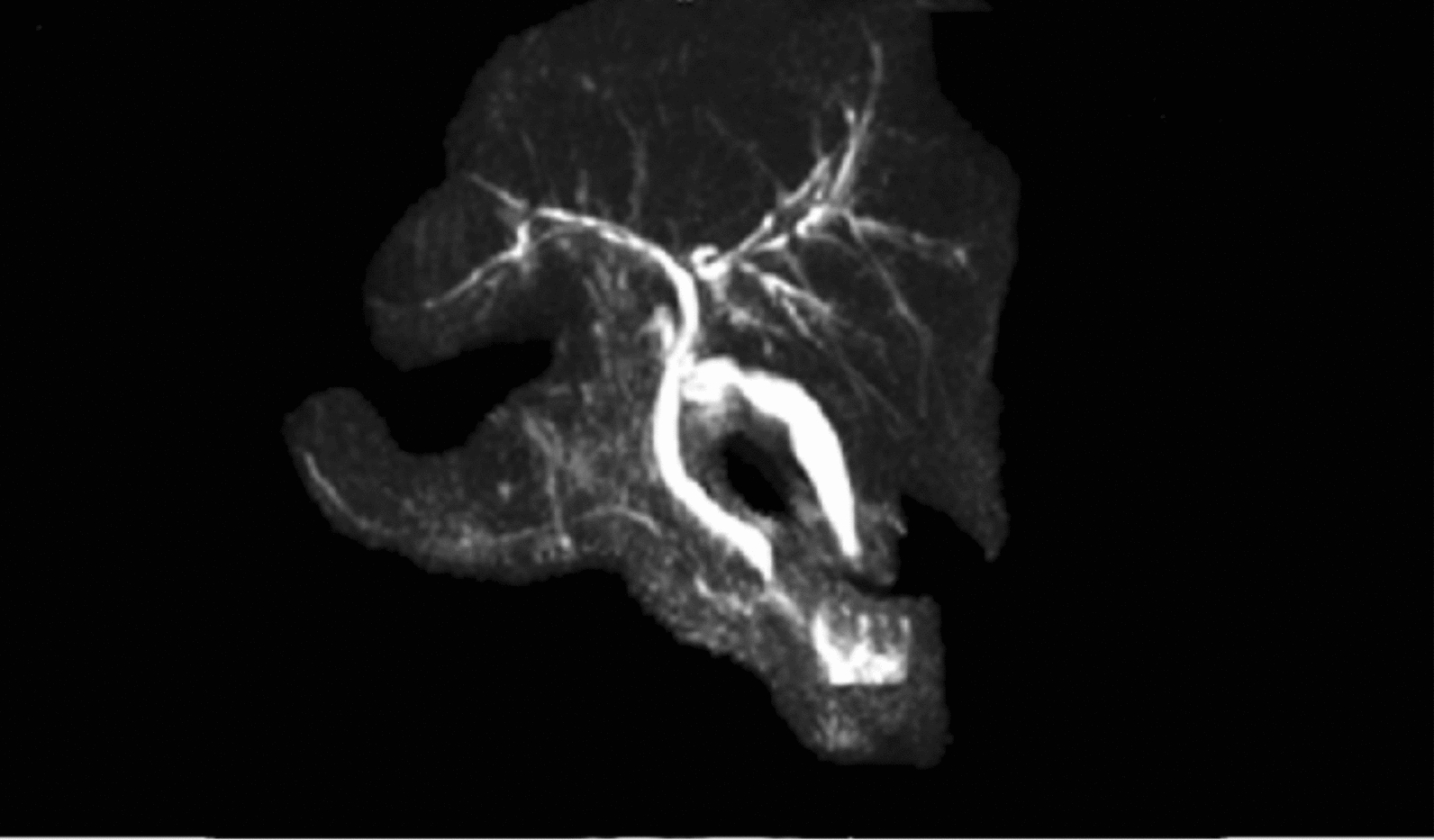
Magnetic resonance cholangiopancreatography demonstrating multiple short-segment biliary strictures, segmental dilatation, and mural irregularities in both intrahepatic ducts

**Fig. 2:**
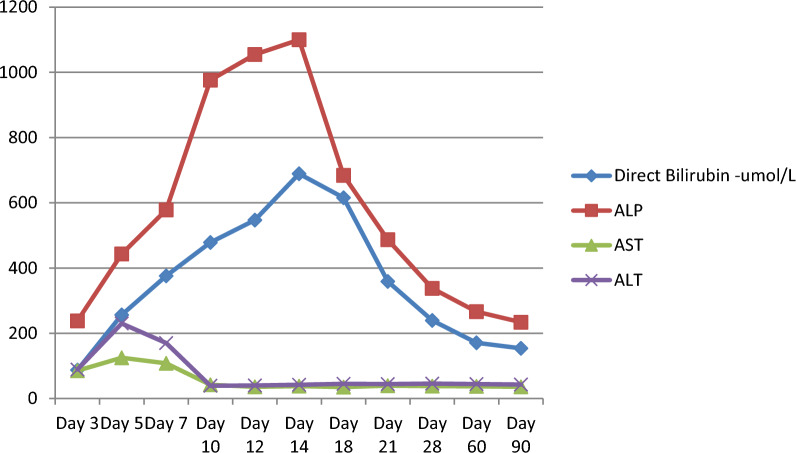
Liver biochemistry of the patient

**Fig. 3:**
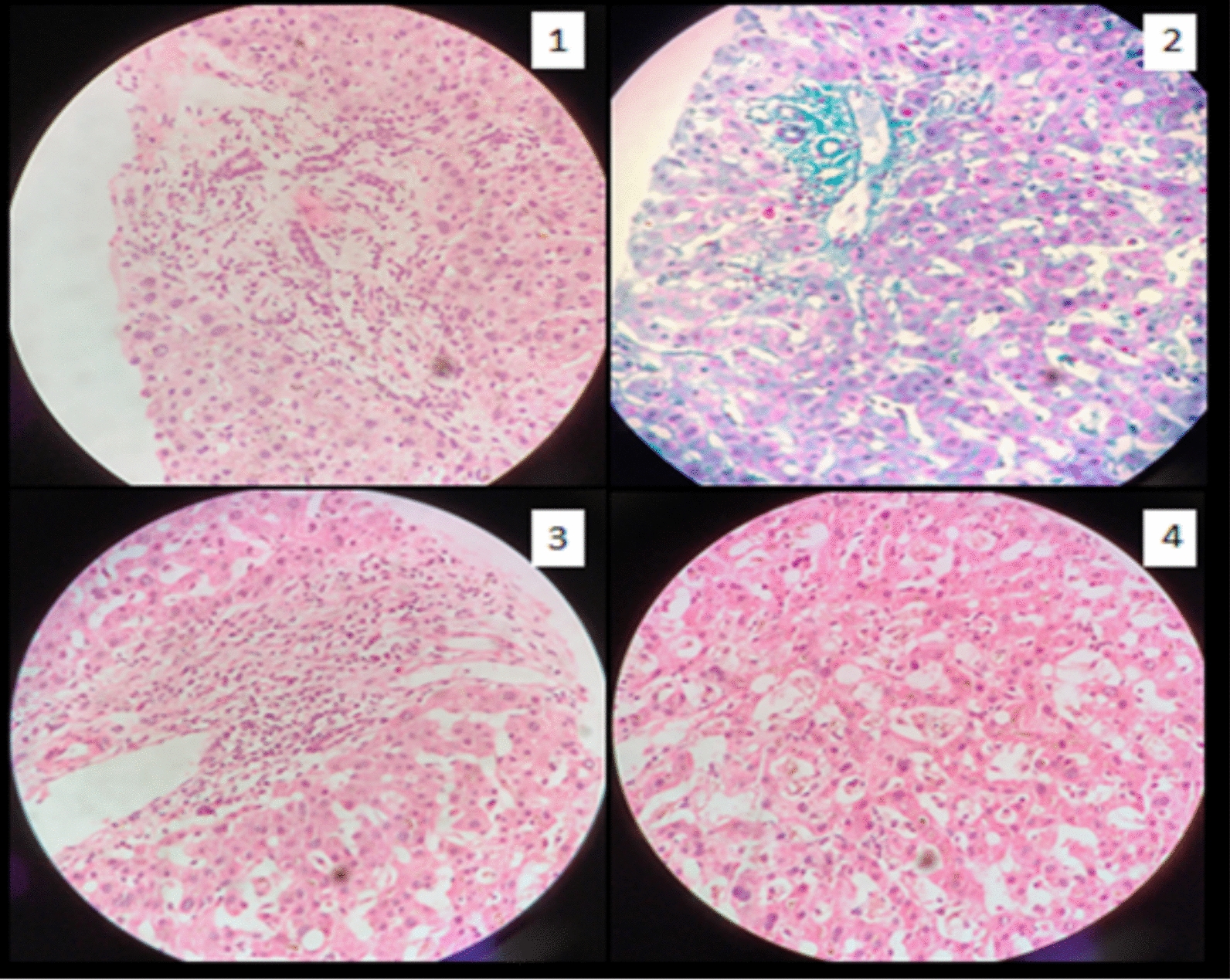
Liver histology—Image 1, 3, 4—hematoxylin and eosin staining (400 magnification) patchy ductular inflammation with lymphocytes, plasma cells, and scattered neutrophils. Image 2: Masson's trichrome stain (400 magnification) panlobular, intracytoplasmic, and canalicular cholestasis present

On magnetic resonance cholangiopancreatography, multiple short-segment biliary strictures, segmental dilatation, and mural irregularities were present in both intrahepatic ducts, confirming the diagnosis of sclerosing cholangitis (Fig. 1). We performed a liver biopsy on day 14 to evaluate the involvement of the small ducts, rule out other potential causes, and rule out overlap syndrome. While awaiting the liver histology report, we offered him a prednisolone 20 mg daily 2-week trial, but he did not show a good rapid clinical or biochemical response to steroids like in autoimmune hepatitis or IgG4 disease (Fig. 2). Patchy ductular inflammation was observed on liver histology (Fig. 3), supporting the diagnosis of sclerosing cholangitis.

The patient did not have a dominant stricture and was not offered an endoscopic retrograde cholangiopancreatography. Over time, the patient experienced clinical improvement and a reduction in cholestatic enzymes (Fig. 2), but the cholestatic enzymes levels did not return to the baseline. The patient was educated on the future need for liver transplantation and followed up in the gastroenterology clinic.

## Discussion

Leptospirosis is a zoonotic disease caused by the spirochete bacteria Leptospira. Globally, leptospirosis diagnoses reach 1.03 million cases annually, resulting in 58,900 deaths [11]. Sri Lanka is considered an endemic region for leptospirosis, with cases reported consistently throughout the year. It has an estimated annual incidence of 52.1 per 100,000 people [12]. The overall median mortality rate of leptospirosis is 2.2%; it significantly increases up to 19% once jaundice develops [13].

Around 66% of cases of leptospirosis experience jaundice, a unique feature that remains incompletely understood. Intrahepatic cholestasis, duodenitis resulting in ampulla of Vater obstruction, sepsis-induced cholestasis, drug-induced liver injury, and indirect hyperbilirubinemia from hemolysis have all been described as causes of jaundice in leptospirosis [14]. Leptospirosis induces direct bacterial invasion of hepatocytes and triggers an inflammatory response by releasing inflammatory cytokines such as TNF- and IL-6. This leads to liver damage either through direct hepatotoxicity or by promoting endothelial dysfunction and microvascular damage within the liver. Leptospira also disrupts intercellular junctions of the hepatocytes, causing bile leakage from the canaliculi into the sinusoidal blood vessels [14].

Sclerosing cholangitis is a rare cause of cholestasis in an infective setting. Infections can damage the epithelial lining of the bile ducts directly, causing inflammation and the release of cytokines and growth factors such as TGF-β. These factors help activate fibroblasts and deposit collagen in the bile ducts [5].Additionally, sepsis triggers the release of toxic, hydrophobic bile, causing damage to the lipid membranes of cholangiocytes and potentially leading to sclerosing cholangitis [15]. These mechanisms are the most likely causes of secondary sclerosing cholangitis in our patient, who has a short history of mild leptospirosis.

The common hepatic artery and its branches solely supply the biliary system, rendering it vulnerable to ischemic damage. The middle third of the common bile duct and the biliary confluence are the most susceptible areas in the biliary system to ischemia. Ischemic cholangiopathy affects the intrahepatic ducts late in the disease [15]. Because intrahepatic bile ducts are affected early in the disease, it is unlikely that our patient had ischemic cholangiopathy leading to secondary sclerosing cholangitisin critically ill patients (SSC-CIP). The other negative features for SSC-CIP were that our patient did not experience septic shock or respiratory distress, nor did he require admission to the intensive care unit for respiratory or cardiovascular support. From the perspective of leptospirosis, our patient had a mild disease, making SSC-CIP unlikely to occur.

Primary sclerosing cholangitis transforms into biliary cirrhosis within 10–15 years of diagnosis, a condition that only a liver transplant can cure [16]. Additionally, 10–20% of people with primary sclerosing cholangitis will eventually develop cholangiocarcinoma [17]. Even though much data are not available, prognosis of the secondary sclerosing cholangitis is worse than primary sclerosing cholangitis, and disease progression is dependent on the treatment of underlying etiology [18]. Failure to properly address the underlying etiology can lead to a rapid progression, ultimately requiring liver transplantation. However, most etiologies of secondary sclerosing cholangitis are not associated with an increased risk of cholangiocarcinoma [19]. It is crucial to diagnose sclerosing cholangitis in a setting of persistent cholestasis and to determine the underlying cause because treating the underlying cause can reduce the rate of progression of sclerosing cholangitis.. Most cases of leptospirosis are diagnosed clinically, involving basic investigations and collateral history. This allows clinicians to start empirical antibiotics while awaiting confirmatory tests, as we did in our patient's case, potentially leading to a favorable outcome.

Despite the patient's severe symptoms related to cholestasis, elevated cholestatic enzymes, and multiple strictures on imaging, we did not offer our patient an endoscopic retrograde cholangiopancreatography and instead treated him medically. We initiated a steroid trial for him primarily to rule out steroid-responsive diseases such as autoimmune hepatitis and IgG4 disease, while we await confirmatory reports. Although we did not observe a rapid clinical and biochemical response to steroids in our patient, the impact of steroids on clinical and biochemical improvement in leptospirosis-induced secondary sclerosing cholangitis remains uncertain, as he experienced clinical and some degree of biochemical improvement over time. Steroids in leptospirosis-induced secondary sclerosing cholangitis are an area that requires further investigation in future research.

Even though the literature does not offer any evident data on follow-up for secondary sclerosing cholangitis patients, we postulated an individualized follow-up plan for our patient.. This included six monthly clinical, biochemical assessments and liver fibrosis evaluations; and annual ultrasound abdomen and osteoporosis screening. If the liver biochemistry is deteriorating, the current plan should include repeating the MRCP and CA 19–9 tests.

## Conclusion

Jaundice, a common finding in leptospirosis, persists even after clinical improvement, leading clinicians to overlook a proper evaluation of this condition. If we fail to properly evaluate jaundice, we may overlook the intriguing complication of secondary sclerosing cholangitis in leptospirosis, which can escalate to cirrhosis. Therefore, it is crucial to monitor the liver functions of patients with leptospirosis even after discharge from inward care.

## Data Availability

No datasets were generated or analysed during the current study.
